# Case Report: Acute intussusception in a 2-year-old male patient: a rare case of Burkitt's lymphoma coexisting with Meckel's diverticulum

**DOI:** 10.3389/fped.2024.1489118

**Published:** 2024-12-05

**Authors:** Xinggui Fang, Biao Yang, Ming Cao, Xiaodong Xu, Benquan Wang

**Affiliations:** ^1^ Department of Pediatric Surgery, The First People’s Hospital of Wuhu, Anhui, China; ^2^ Department of Pathology, The First People’s Hospital of Wuhu, Anhui, China; ^3^ Department of Ultrasonics, The First People’s Hospital of Wuhu, Anhui, China; ^4^ Department of Gastrointestinal Surgery, The First People’s Hospital of Wuhu, Anhui, China

**Keywords:** intussusception, Burkitt’s lymphoma, acute abdominal pain, Meckel’s diverticulum, child

## Abstract

In children, 90% cases of intussusception are idiopathic and the remaining 10% are attributed to underlying diseases (most commonly due to Meckel's diverticulum, polyps then either duplication cyst or mesentery cysts, and rarely due to Burkitt's lymphoma). The occurrence of acute intestinal intussusception caused by Burkitt's lymphoma in children under the age of 5 is exceedingly rare. Burkitt's lymphoma presents with diverse clinical manifestations, often leading to the identification of an abdominal tumor in pediatric patients. This highly aggressive and rapidly proliferating neoplasm can induce indirect symptoms due to compression or direct involvement of the intestinal lumen, resulting in intussusception. Herein, we present a case report of ileocolic-type intussusception in a 2-year-old boy, which was attributed to the coexistence of Burkitt's lymphoma and Meckel's diverticulum. Notably, this patient exhibited atypical clinical features for Burkitt's lymphoma and did not belong to the high-risk demographic associated with this rare disease. Furthermore, this case represents a unique combination involving the most prevalent cause of Meckel's diverticula and the rarest etiology of Burkitt's lymphoma.

## Introduction

Acute intussusception is a prevalent cause of acute abdominal pain in pediatric patients (1). The primary clinical manifestations include intermittent abdominal pain, vomiting, hematochezia, and the presence of an abdominal mass (2). Although the etiology of acute intussusception in children remains poorly understood, approximately 90% of cases are considered idiopathic without any identifiable organic disease (3). However, underlying causes can be identified in about 10% of cases, such as Meckel's diverticulum (MD), polyps, duplicated segments, mesenteric cysts, and lymphoma (4). Burkitt's lymphoma (BL) is an exceptionally rare cause observed primarily in children under 5 years old (5, 6). Non-Hodgkin lymphomas are the most prevalent extranodal lymphomas, with the ileum being the primary site for lymphoma occurrence due to its abundant gut-associated lymphoid tissue. Histologically, BL is characterized by a distinctive “starry sky” appearance, indicative of its high mitotic and apoptotic activity. Despite diagnostic challenges, children with completely resected early-stage (I or II) Burkitt lymphoma have shown promising treatment outcomes through multiagent chemotherapy agents, achieving a 4-year event-free survival (EFS) rate of 98% and a 4-year overall survival rate (OS) of 99%. In this report, we present a case study involving the youngest Chinese boy diagnosed with acute intussusception due to BL and MD.

## Case presentation

**Figure 1:**
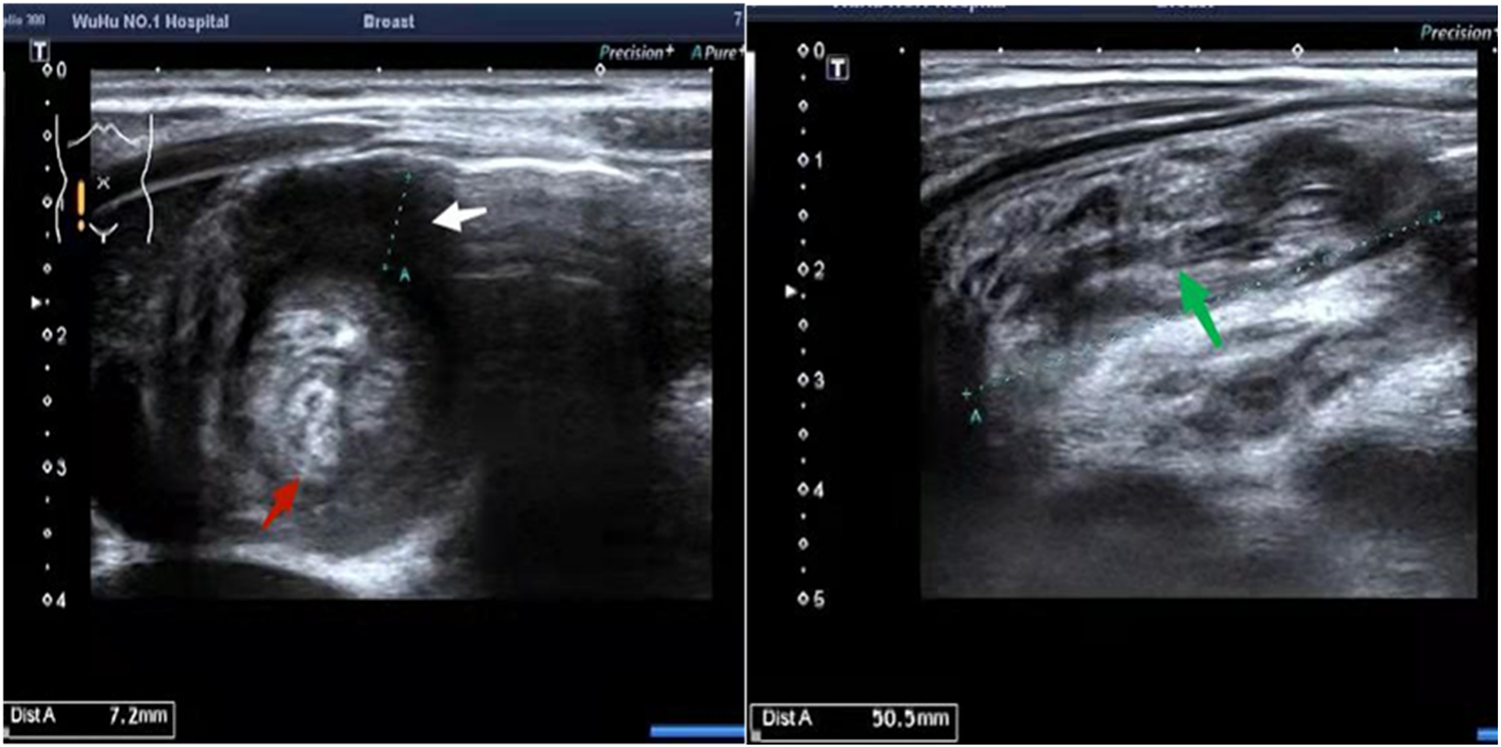
Abdominal ultrasonography reveals the presence of the concentric circle sign in the transverse section (indicated by red and white arrows) and the sleeve sign in the longitudinal section (indicated by a green arrow).

The emergency room received a 2-year-old Chinese boy on May 7, 2022, presenting with a 1-day history of abdominal pain. The patient exhibited no fever, vomiting, bloody stools, constipation or other gastrointestinal symptoms. Upon admission, the patient's overall condition was satisfactory and all vital signs fell within normal ranges. The patient measured 85 cm in height and weighed 12.5 kg. Abdominal palpation revealed the presence of a mass measuring approximately 40 × 50 mm in the right upper abdomen region. Bowel sounds were noted to be hyperactive. Abdominal ultrasonography indicated concentric circle sign in transverse section, sleeve sign in longitudinal section and abnormal mass echoes within the intussusception loop (Figure 1).

The laboratory test results revealed the following findings: the white blood cell count was 12.6 × 10^9^/L (above the normal range of 4–9.5 × 10^9^/L), with a neutrophil percentage of 87.8% (above the normal range of 55%–75%). The hemoglobin level was measured at 103 g/L (above the normal range of 130–170 g/L), and C-reactive protein (CRP) level was elevated at 18 mg/L (above the normal range of 0–5 mg/L). Blood biochemical analysis indicated an increased uric acid (UA) value of 512 umol/L (above the normal range of 142–440 u/L) and lactate dehydrogenase (LDH) values were elevated to 342 u/L (above the normal range of 80–285 umol/L). Other results were within normal limits.

**Figure 2:**
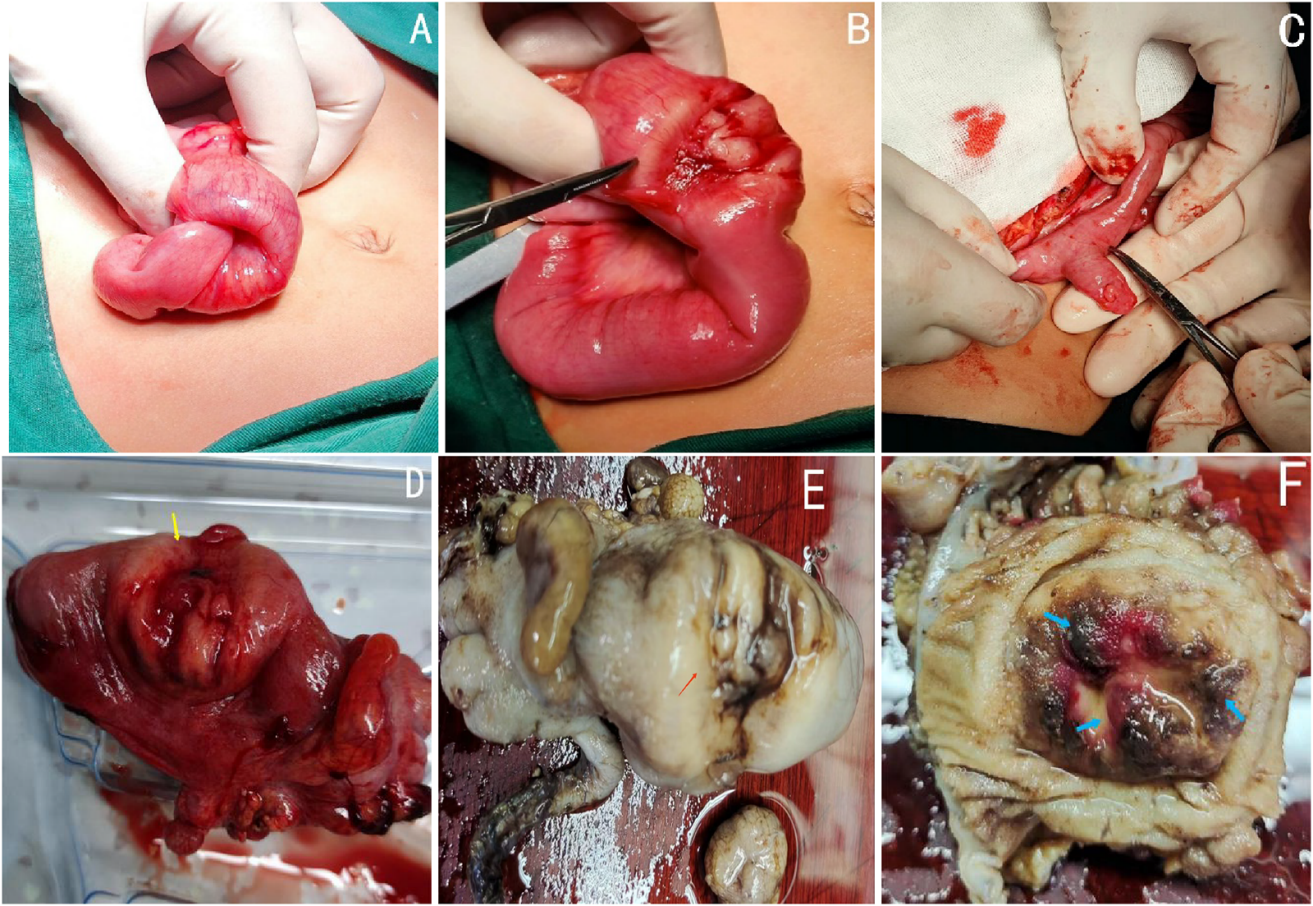
Intraoperative view of intussuscepted segment of bowel **(A)**. After manual reduction, two lesions were identified at distances of 3 cm and 8 cm from the ileocecal valve, respectively (**B**: terminal ileal tumor; **C**: MD). Surgically excised specimens, including the terminal ileum, cecum, and a segment of the ascending colon **(D)**. Gross view of the formalin-fixed ileocecum specimen showed the tumor invading ileocecal valves (**E**,**F**, the blue arrows).

The patient initially underwent ultrasound-guided hydraulic enema reduction, which proved unsuccessful. Due to the failure of hydrostatic enema reduction, an exploratory laparotomy was performed through a transverse incision of the right side of the abdomen. During the operation, ileo-colonic intussusception measuring approximately 10 cm in length was identified at the terminal ileum (Figure 2A). Successful manual reduction of the intussuscepted bowel was achieved; however, post-reduction examination revealed two lesions located at the distal end of the intussuscepted ileum: an intestinal wall mass positioned 3 cm away from the ileocecal valve and a Meckel's diverticulum situated 8 cm away (Figures 2B,C). The sizes of these lesions were measured as 4 × 3 cm and 2 × 3 cm respectively. The mass within the intestinal wall encompasses approximately 75% of the circumference of the intestinal canal, exhibiting infiltrative growth and severe thickening of the serosa. The central region displays a slightly depressed morphology resembling that of a “volcano”, while no signs of hemorrhage, purulence or other abnormalities were observed in Meckel's diverticulum. Based on the growth site and morphological characteristics of the intestinal wall mass, a highly probable diagnosis of malignancy was suggested following differential analysis of diseases in this region. To achieve a negative surgical margin, the distal ileum, cecum, and proximal ascending colon were excised with consideration of the close proximity (approximately 3 cm) to the ileocecal valves (Figure 2D). Subsequently, an ileocolon anastomosis was performed to restore continuity. Additionally, a wedge resection was conducted on the MD. Upon formalin fixation, subsequent dissection of the specimen in the ileocecal region revealed tumor infiltration into the ileocecal valves (Figures 2E,F, blue arrows).

**Figure 3:**
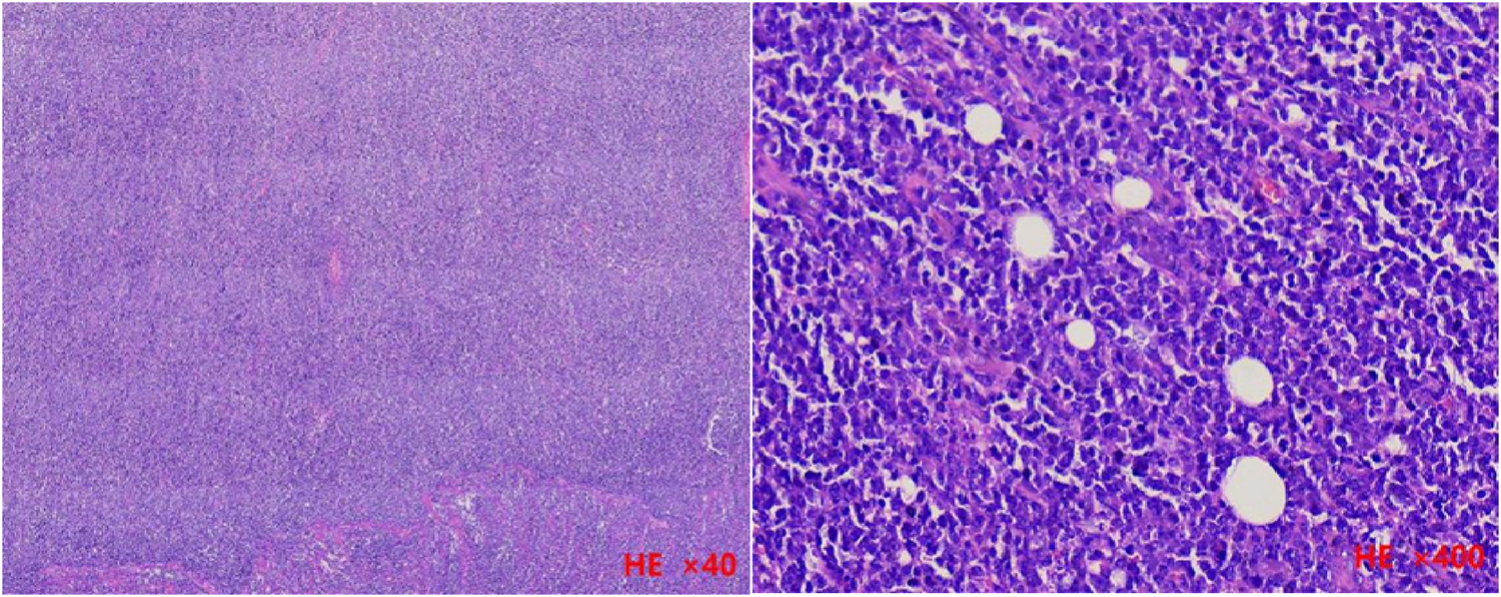
The histopathological examination of the resected tissue revealed tumor cell infiltration throughout all layers of the intestinal wall in a low-magnification view (H&E stained, ×40). Additionally, a high-magnification view (H&E stained, ×400) demonstrated numerous mitotic figures, apoptotic cells, and scattered macrophages containing apoptotic bodies, giving rise to a characteristic “starry sky” phenomenon.

The histopathological examination of the excised mass revealed a full-thickness infiltration of lymphoid cells interspersed with scattered tingible body macrophages, exhibiting a “starry sky” phenomenon (Figure 3).

**Figure 4:**
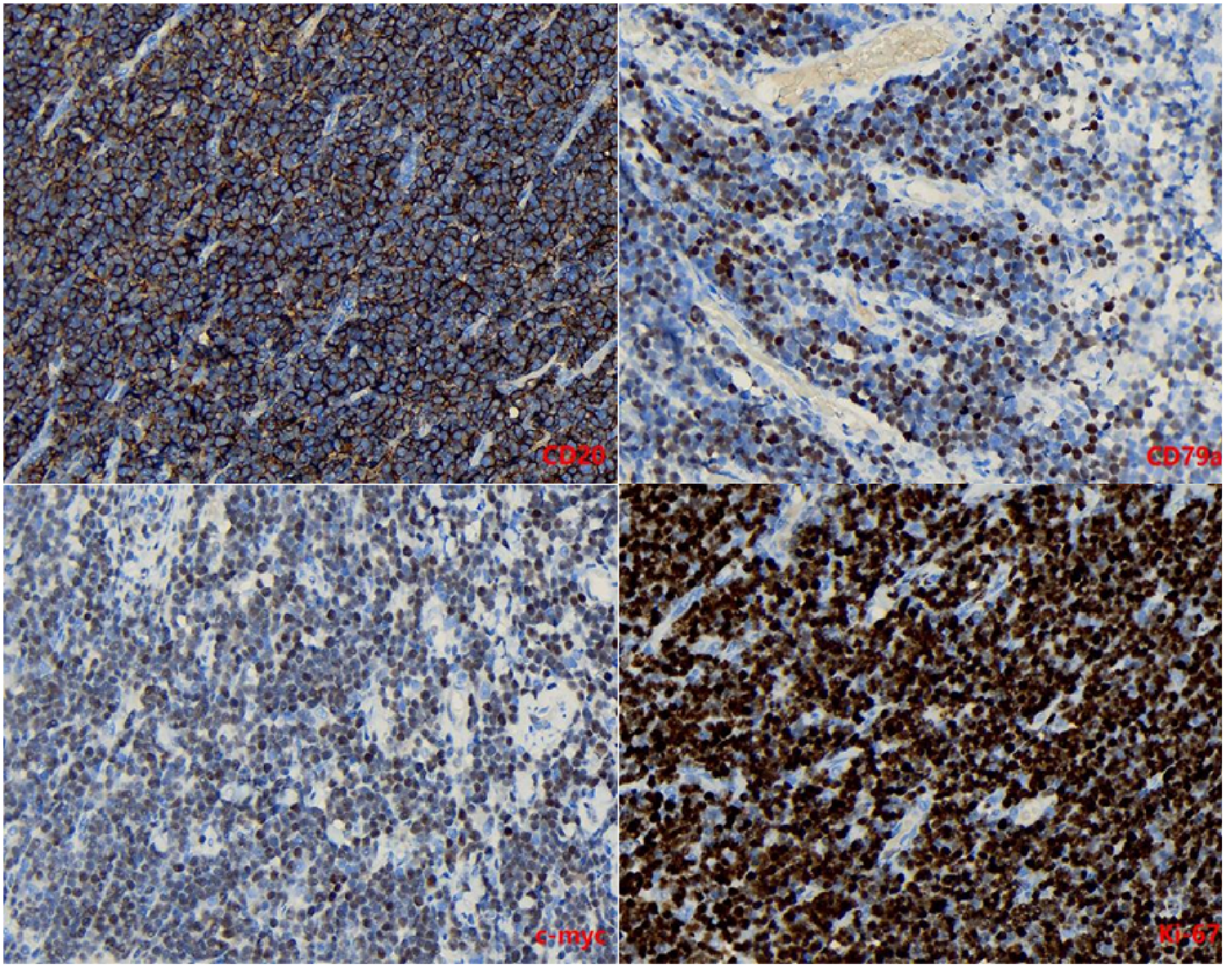
Immunostaining for CD20, CD79a, c-myc was positive (×400). The Ki-67 labeling index was close to 100% (×400).

The immunostaining results for CD20, CD79a, and c-myc were positive, while the Ki-67 labeling index approached 100% (Figure 4).

The patient was ultimately diagnosed with acute intussusception, resulting from intestinal Burkitt's lymphoma and Meckel's diverticula. He made a satisfactory recovery following the surgical procedure and was subsequently transferred to the oncology hospital for chemotherapy after 2 weeks. The patient was diagnosed with stage II Burkitt's lymphoma and subsequently referred for evaluation and initiation of chemotherapeutic treatment. He received two cycles of CHOP (cyclophosphamide, vincristine, prednisone, and doxorubicin) at an oncology hospital. During the 2-year follow-up period, no instances of tumor recurrence or metastasis were observed.

## Discussion

The term “intussusception” refers to the telescopic displacement of the proximal segment of the intestine into the lumen of the adjacent distal segment. It is commonly observed in infants and young children. Approximately 85% of intussusceptions occur in children aged between 6 months and 2 years, with 75% presenting specific symptoms. The classic triad of intussusception includes abdominal pain, abdominal mass, and bloody stools. In contrast to children, adults with intussusception often exhibit nonspecific clinical manifestations such as chronic abdominal pain, abdominal mass, intestinal obstruction, or weight loss. In adults, 90% of cases are associated with intestinal neoplasms; however, this percentage is less than 1% in young children (7). Abdominal ultrasound is considered the most appropriate diagnostic examination for suspected intestinal intussusception in children as it enables a positive diagnosis when combined with typical clinical symptoms (8).

The etiology of intussusception in children is predominantly idiopathic; however, approximately 10% of cases are associated with an underlying cause, such as Meckel's diverticulum (MD), polyp, duplication, mesenteric cyst, or lymphoma. MD is a relatively common cause of acute intussusception, whereas Burkitt's lymphoma (BL) is an exceedingly rare cause accounting for only 3%–5% and 0.4%–1% of cases respectively (9). BL, classified as one of the most prevalent types of lymphoma, represents a highly aggressive form of non-Hodgkin lymphoma (NHL) that can be categorized into endemic, sporadic, and immunodeficiency-associated subtypes (10). Sporadic BL primarily manifests as abdominal or other organ-specific lymphomas and frequently occurs in the ileum and colon due to their high concentration of lymphoid tissue (11). The predisposing factors for sporadic BL do not exhibit significant correlations with geography or climate conditions; however, they are closely associated with infections such as EBV, HHV-8, HIV, and HTLV-1, familial predisposition, and exposure to ionizing radiation. All these risk factors were absent in our medically free patient. Characterized by early onset (27% aged 3–5 years; 25% aged 6–9 years; 6% aged 0–2 years; average age: 7.8 years), BL exhibits a higher prevalence among males than females.

Acute intussusception is a rare clinical presentation of sporadic Burkitt lymphoma (BL), and its co-occurrence with Meckel's diverticulum (MD) is even rarer. To the best of our knowledge, there have been no reports documenting acute BL-associated intussusception in children under 2 years old or any reports describing intussusception caused by Burkitt lymphoma combined with MD, both domestically and internationally. Although several cases of childhood intussusception caused by BL have been reported worldwide in the past, these cases typically share common features such as chronic abdominal pain, abnormal blood findings or abdominal mass, and occur in children older than 5 years (12, 13). This case highlights two notable aspects: firstly, it presents the initial documented occurrence of acute intestinal Burkitt's lymphoma resulting in intussusception in a 2-year-old child; secondly, the etiology of this condition may involve both Burkitt's lymphoma and Meckel's diverticulum (MD), which is exceptionally rare in clinical practice. While our final conclusion attributes primary causality to Burkitt's lymphoma, MD incidentally plays a supplementary role through pathological diagnosis. Notably, immunostaining for Ki-67 labeling index revealed nearly 100% positivity, confirming the highly aggressive nature of BL. Nevertheless, due to the potential for complete tumor resection, the prognosis for Burkitt's lymphoma presenting with acute intussusception remains favorable (14).

Additionally, it is likely that MD was not the primary cause of intussusception in this case; however, we hypothesize that MD may have indirectly contributed to this condition through its potential effect on bowel motility (15, 16). The occurrence of a disease resulting from both the most common and least common causes simultaneously is exceedingly rare in clinical practice, and the manifestation of this disease at an atypical age further highlights its unique nature. Ultimately, the patient achieved a favorable outcome due to a combination of surgical intervention and chemotherapy agents. Timely diagnosis and treatment significantly contribute to a positive prognosis.

## Data Availability

The datasets presented in this article are not readily available. Requests to access the datasets should be directed to 576729917@qq.com.
